# miR-155 induction is a marker of murine norovirus infection but does not contribute to control of replication *in vivo*

**DOI:** 10.12688/wellcomeopenres.14188.1

**Published:** 2018-04-18

**Authors:** Lucy Thorne, Jia Lu, Yasmin Chaudhry, Ian Goodfellow

**Affiliations:** 1 Division of Virology, Department of Pathology, University of Cambridge Addenbrooke's Hospital Cambridge, Cambridge, CB2 0QQ, UK; 2 Division of Infection and Immunity, University College London, London, WC1E 6BT, UK

**Keywords:** norovirus, MNV, microRNA, miR-155, persistent infection

## Abstract

**Background:** Due to their role in fine-tuning cellular protein expression, microRNAs both promote viral replication and contribute to antiviral responses, for a range of viruses. The interactions between norovirus and the microRNA machinery have not yet been studied. Here, we investigated the changes that occur in microRNA expression during murine norovirus (MNV) infection.

**Methods:** Using RT-qPCR-based arrays, we analysed changes in miRNA expression during infection with the acute strain MNV-1 in two permissive cell lines, a murine macrophage cell line, RAW264.7, and a murine microglial cell line, BV-2. By RT-qPCR, we further confirmed and analysed the changes in miR-155 expression in the infected cell lines, bone-marrow derived macrophage, and tissues harvested from mice infected with the persistent strain MNV-3. Using miR-155 knockout (KO) mice, we investigated whether loss of miR-155 affected viral replication and pathogenesis during persistent MNV-3 infection in vivo and monitored development of a serum IgG response by ELISA.

**Results: **We identified cell-specific panels of miRNAs whose expression were increased or decreased during infection. Only two miRNAs, miR-687 and miR-155, were induced in both cell lines. miR-155, implicated in innate immunity, was also upregulated in bone-marrow derived macrophage and infected tissues. MNV-3 established a persistent infection in miR-155 knockout (KO) mice, with comparable levels of secreted virus and tissue replication observed as for wildtype mice. However, serum anti-MNV IgG levels were significantly reduced in miR-155 KO mice compared to wildtype mice.

**Conclusions:** We have identified a panel of miRNAs whose expression changes with MNV infection. miR-155 induction is a marker of MNV infection in vitro and in vivo, however it does not contribute to the control of persistent infections in vivo. This finding suggests that the immune defects associated with miR-155 deletion, such as lower serum IgG levels, are also not important for control of persistent MNV-3 infection.

## Introduction

Noroviruses belong to the *Caliciviridae* family of small, positive strand RNA viruses. Human noroviruses (HuNoVs) are a major cause of viral gastroenteritis worldwide, causing around 2–3 million infections in the UK every year ^1^. The majority of infections are acute and occur in large outbreaks that sweep through places with close living environments, such as hospitals, at a considerable cost to the NHS ^2^. Although mortality rates are generally low, immunocompromised individuals are highly susceptible to HuNoV, often developing chronic infections, which represent a significant burden of morbidity in transplant recipients ^3^.

Until very recently, HuNoVs had remained refractory to all attempts to establish a cell culture system. The recent demonstration of HuNoV replication in enteric organoids ^4^ and a cultured B cell line ^5^ represent significant breakthroughs. However, understanding of norovirus replication and in particular its interactions with the host cell currently still lags behind that of other viruses. As an alternative model system, murine norovirus (MNV) has been widely used to characterise norovirus replication. Like HuNoV, it is an enteric virus, which transmits by the faecal-oral route, and it has been found in wild and laboratory mice ^6, 7^. It remains the only norovirus that replicates in cell culture to produce high titres of infectious virus and has a tropism for macrophage, dendritic cells, B and T cells, with a recent study also identifying a role of enterocytes *in vivo* ^5, 6, 8, 9^. This, combined with the availability of reverse genetics systems ^10– 12^ and strains that cause both acute and persistent infections in the natural host ^6, 11^, makes MNV a valuable model for characterising norovirus-host interactions.

MicroRNAs (miRNAs) are short non-coding RNAs, which post-transcriptionally silence over 50% of human mRNAs to fine-tune protein expression, contributing to the regulation of diverse cellular processes ^13, 14^. Interactions with the cellular miRNA repertoire have been shown for a diverse range of viruses, including RNA, DNA, acute and persistent viruses. miRNAs have been reported to either promote or inhibit viral replication, in general by indirectly regulating expression of cellular cofactors or the immune response respectively ^15, 16^, although direct interactions with viral RNAs have also been reported ^17, 18^. As such a number of viruses actively manipulate cellular miRNA levels, either specifically or by targeting the miRNA processing machinery to initiate a global shutdown in miRNA biogenesis ^19– 21^. Identifying the changes in miRNA expression that occur with infection can reveal new proteins or pathways that play a role in viral lifecycles.

To date there have been no reports on the interaction between noroviruses and the cellular miRNA machinery. Here, we aimed to determine whether MNV infection causes changes in miRNA expression in permissive cell lines, and whether any of the observed effects contribute to viral pathogenesis and affect the outcome of infection *in vivo*.

## Methods

### Cells

Murine macrophage cell lines RAW264.7 and microglial cell line BV-2 (provided by Jennifer Pocock, University College London) were maintained in Dulbecco’s modified Eagles Medium (DMEM, Gibco) containing 10% fetal calf serum (FCS), penicillin (P) (100 SI units/mL) and streptomycin (S) (100 μg/mL) and 10 mM HEPES (pH7.6) at 37°C with 10% CO _2_. For virus recovery BHK cells engineered to express T7 RNA polymerase (BSR-T7 cells, obtained from Karl-Klaus Conzelmann, Ludwid Maximillians University, Munich, Germany) were maintained in DMEM containing 10% FCS, penicillin (100 SI units/mL) and streptomycin (100 μg/mL) and 0.5 mg/mL G418. For preparation of bone marrow derived macrophage (BMDM), bone marrow cells were harvested from female C57BL/6 mice and were cultured in DMEM containing 10% fetal calf serum (FCS), penicillin (100 SI units/mL) and streptomycin (100μg/mL). For differentiation the supernatant from CMG14 cells, which contains macrophage colony stimulating factor (M-CSF), was added to the media for 5–7 days.

### Virus recovery and infection

Recombinant viruses were rescued from cDNA clones containing either the MNV-1 ^10^ or MNV-3 genomes ^11^ using the reverse genetics system based on recombinant Fowlpox expressing T7 RNA polymerase, as previously described ^10^. Fifty percent tissue culture infectious dose (TCID50) titrations were performed on RAW264.7 and BV-2 cells. For miRNA analysis, cell lines were infected with an acute strain of MNV (MNV-1) at an MOI of 0.1 TCID50/cell, or were mock infected. BMDM were infected with MNV-1 at an MOI of 10 TCID50/cell (as determined in RAW264.7 cells).

### microRNA extraction and analysis

To analyse miRNA expression the small cellular RNA fraction (less than 200nt) was harvested at 20 hpi from cell lines, and 24 hpi from BMDMs, using the miRVana RNA isolation kit, as per manufacturer’s instructions. For cDNA synthesis the Taqman miRNA reverse transcription kit was used (Life Technologies), according to manufacturer’s instructions. 1000 ng of RNA extracted from infected and uninfected RAW264.7 cells and BV-2 cells were used with Megaplex RT primers, Rodent Pool A (Life Technologies). The pool contains primers specific to 335 and 238 mature unique mouse and rat miRNAs respectively, alongside primers for 4 specific endogenous controls. The cDNA was then used for qPCR using Taqman Rodent miRNA Array A cards (TDLA, Life Technologies, as per manufacturer’s instructions), which contain primer-probe sets specific for 381 rodent miRNAs. The TLDA cards were run on the 7900HT T Fast Real-Time PCR System and the data was visualised and analysed for the Ct value of each miRNA by RQ manager software (Life Technologies). Further analysis was performed using Microsoft Excel. The Ct value for each miRNA was normalised against the Ct value for the endogenous control small nuclear RNA, U6, which did not change with infection. A two-fold change was used to indicate a significant change, as suggested in the manufacturer’s instructions. RT-qPCR was also performed using individual RT and PCR primer-probes specific to miR-155.

### *In vivo* studies

For analysis of miR-155 expression, three-to-four week old female C57BL/6 mice were inoculated with 1000 TCID50 of MNV-3 by oral gavage. Each group contained 3 mice and a control group was mock infected. The mesenteric lymph node (MLN), caecum and colon were isolated on day 2 post infection (dpi) for RNA extraction. miR-155 knock out mice (Jackson Laboratories) and wild type control C57BL/6 mice were inoculated with 10 TCID50 of MNV-3 by oral gavage, with 5 mice in each group. Mice were weighed and faecal samples were collected on 1–7, 14 and 21 dpi. Serum samples were collected on days 0 ,7, 14 and 21 pi, and mice were euthanized on day 21 pi. Tissue samples were harvested from separate groups on day 2 post infection.

### RNA extraction and RT-qPCR for viral genome copies

Tissues were homogenised into RNA lysis buffer using ceramic beads (BioSpec Products) with a Fast Prep-24 homogeniser (MP Biomedicals). Faecal pellets were homogenised in PBS (100mg/mL), followed by centrifugation at 4000 rpm for 5 min, 4°C. RNA was extracted from 100 μL of supernatant using the GenElute total mammalian RNA kit (Sigma). Quantification of viral genome copies was performed by two-step RT-qPCR, with reverse transcription using with M-MLV RT (Promega) with random hexamers, as per manufacturer’s instructions. qPCR was then performed on the cDNA using a Taqman Low Rox qPCR mastermix (Primer design), with primers (MNV-3 F: CCGCAGGAACGCTCAGCAG and R: GGCTGAATGGGGACGGCCTG), and probe (ATGAGTGATGGCGCA). The Viia ^TM^ 7 Real time PCR machine was used with an initial denaturation step of 8 min at 95°C, followed by 50 cycles of 95°C for 10 s and 60°C for 1 min. The genome copy number was interpolated from the standard curve and was calculated per ng of RNA or per mg of stool depending on the sample, using Microsoft Excel. All graphs were produced using Graph Pad Prism V.5 Software.

### ELISA for serum anti-MNV IgG

We performed ELISAs for detecting MNV-specific serum IgG in peripheral blood as previously reported (Hwang *et al*., 2014, Wobus *et al*., 2004). Briefly, MNV-3 virus-like particles (VLPs), kindly provided by Stephanie Karst (University of Florida, Gainsville, USA) were diluted 1:100 using carbonate buffer at pH 9.6 and 50 μl of VLPs were used per well to coat the Nunc MaxiSorp™ 96-well plate overnight at 4°C. Serum was collected as the supernatant after spinning the whole blood at 15,000 x g for 5 minutes at 4°C and was used at 1:100 dilution in 50 μl total volume. The reactions were developed by adding 100 μl 1-Step™ Turbo TMB-ELISA Substrate Solution (Life Technologies) and stopped by adding equal volume of 1N sulphuric acid solution. The absorbance at 450 nm were read and normalised absorbance were calculated by subtracting the mean absorbance of wells without primary antibody. The cutoff of positive results was determined as the mean + 3 × standard deviation of mock serum absorbance.

## Results

**Figure 1.**
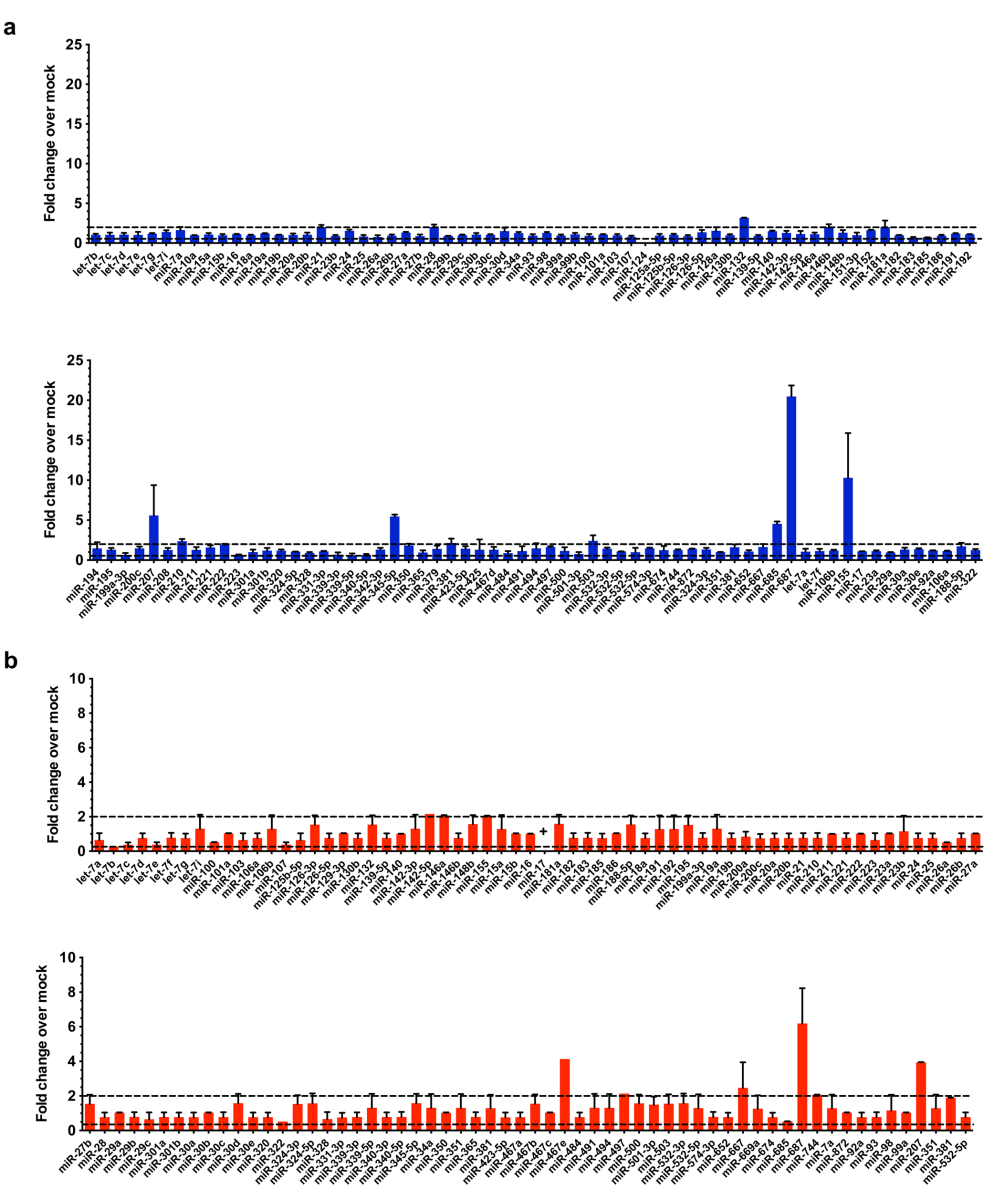
Changes in miRNA expression with MNV-1 infection in two permissive cell lines. ( **a**) Murine macrophage RAW264.7 cells ( **b**) murine microglial BV-2 cells were infected with MNV-1 at an MOI of 0.1 TCID50/cell. The small RNA fraction was harvested at 20 hpi. Reverse transcription was performed using a set of primers specific for 380 miRNAs, followed by qPCR analysis using TLDA miRNA cards. The relative quantity value on the y-axis is equivalent to fold change. The dashed lines indicate a 2-fold increase and decrease, above or below which the change is considered significant.

To investigate changes in miRNA expression with norovirus infection, we infected two permissive cell lines, a murine macrophage cell line, RAW264.7, and a murine microglial cell line, BV-2, with an acute strain of MNV (MNV-1). We analysed miRNA expression at 20 hours post infection (hpi), a time selected to coincide with the peak in innate immune responses against MNV-1 previously identified ^22^, as many macrophage miRNAs are thought to be involved in regulating interferon (IFN) responses ^23^. We found that only a small panel of miRNAs had altered expression as a result of MNV-1 infection in both RAW264.7 and BV-2 cells, whereas the majority did not change by more than 2-fold ( Figure 1A). In total 6 miRNAs were upregulated by more than 2-fold in RAW264.7 cells: miR-687 (20.5x), miR-155 (10.5x), miR345-5p (5.5x), miR-658 (4.5x), miR-132 (3.1) and miR-210 (2.6x). In contrast, 5 miRNAs were downregulated in infected BV-2 cells: miR-let7b (4.0x), miR-207 (3.9x), miR-146a and miR-744 (both 2.1x), and miR-17 which was highly expressed in uninfected cells and decreased to below the level of detection with infection, representing the greatest change in BV-2s ( Figure 1B). Taken together these results indicate cell-specific responses to viral infection. The only common response was the induction of miR-687 and miR-155, which were also upregulated in both cell lines but to a lesser extent in BV-2 cells than in RAW264.7 cells (6.1x and 2.0x respectively), ( Figure 1B).

**Figure 2.**
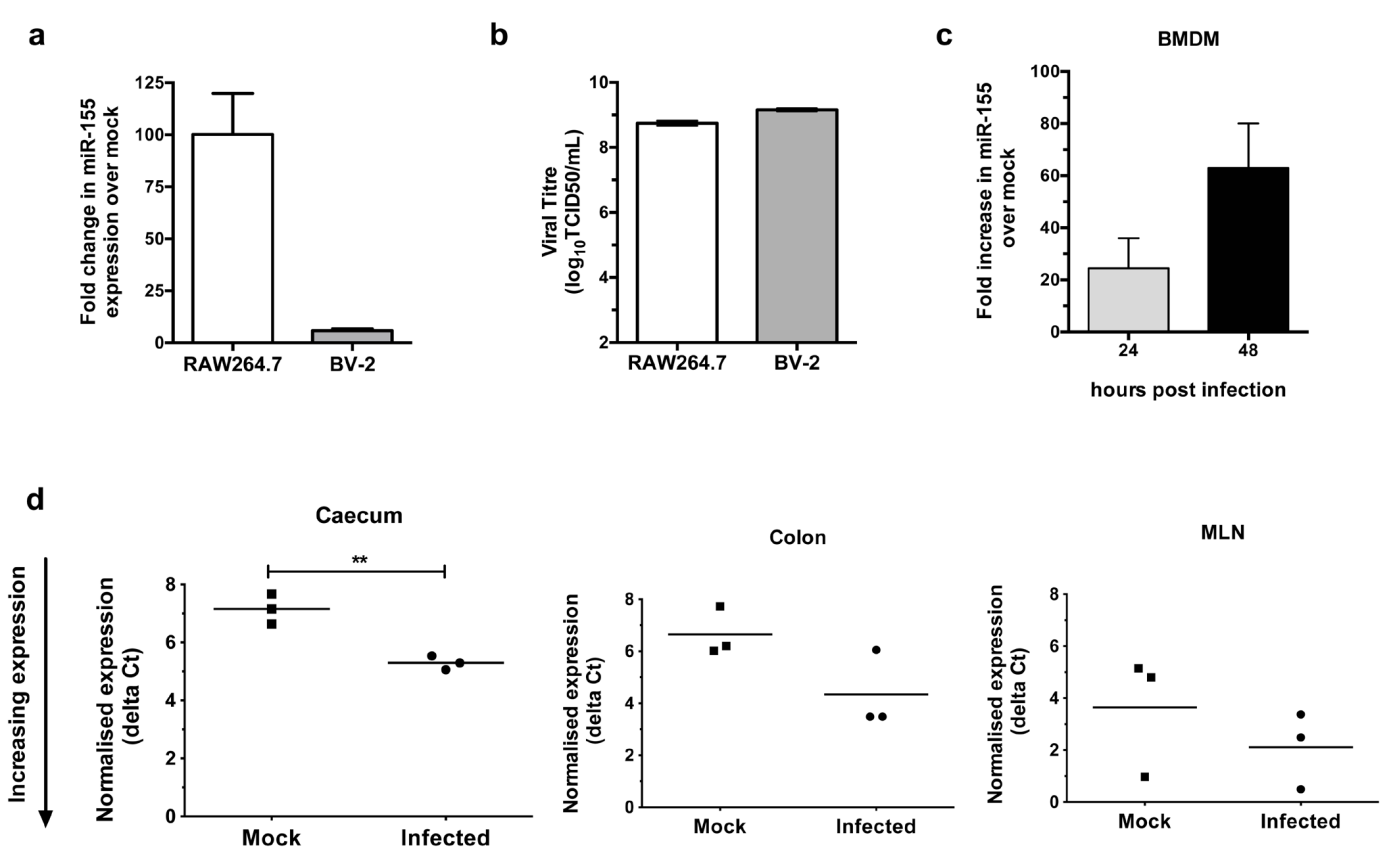
miR-155 is upregulated in MNV-infected cells and tissues. ( **a**) The fold change in miR-155 mature transcript levels with MNV-1 infection in RAW254.7 cells and BV-2s (0.1 TCID50/cell) at 20 hpi. ( **b**) MNV-1 replicates to similar levels in RAW264.7 cells as BV2 cells. Viral titres were determined by TCID50. ( **c**) miR-155 is upregulated over a time course of infection of MNV-1 in BMDMs. ( **d**) miR-155 expression is induced in tissues infected with MNV-3 harvested at day 2 post infection from wildtype mice.

miR-155 is one of the most well-characterised miRNAs, with known links to innate immunity and an antiviral response ^24, 25^. We therefore focused on miR-155, hypothesising that the increase in miR-155 may contribute to or potentially regulate the antiviral response to MNV infection. We first validated miR-155 induction in infected RAW264.7 and BV-2 cells using RT-qPCR. Compared to the data obtained using qPCR-based arrays, we detected an even greater increase in miR-155 expression in both infected cell types at the same time point post infection, with an induction of 100x in RAW264.7 cells ( Figure 1A and Figure 2A). Also in line with the array data, miR-155 was more highly induced in infected RAW264.7 cells compared to infected BV-2s ( Figure 2A), despite MNV-1 attaining similar levels of replication in both cell types ( Figure 2B). We further validated the induction of miR-155 in MNV-1-infected bone-marrow derived macrophage cells (BMDMs). miR-155 expression was induced approximately 20-fold at 24 hpi in BMDMs, which rose to approximately 60-fold at 48 hpi ( Figure 2C).

To determine whether miR-155 is upregulated during MNV infection *in vivo*, we infected immunocompetent mice with the persistent MNV-3 strain, and harvested tissues at day 2 post infection. This timing coincides with the peak in viral loads during the acute phase of infection, when virus can be detected in the caecum, colon and mesenteric lymph node (MLN) ^11^. We found that miR-155 was significantly increased in the caecum, the primary site for MNV replication ^11^, and a trend for induction was observed in the colon and MLN, although this was not significant.

**Figure 3.**
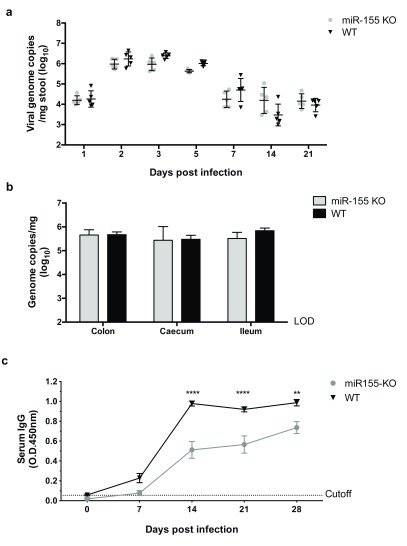
MNV-3 persistence and replication is not affected in miR-155 KO mice. ( **a**) MNV-3 is secreted at similar levels in the faeces from miR-155 KO mice as WT mice over the course of a persistent infection. ( **b**) MNV-3 dissemination and replication in the tissues was comparable in miR-155 KO mice and WT mice at day 2 post infection. LOD indicated limit of detection. ( **c**) miR-155 KO mice have impaired serum anti-MNV IgG levels compared to WT mice.

To determine whether miR-155 contributes to an antiviral response, we infected wildtype (WT) and miR-155 knockout (KO) mice with MNV-3. We choose to assess the role of miR-155 in a persistent model of infection as a recent study showed that miRNAs play a greater role in chronic infections than in acute infections, through regulating the expression of pro-inflammatory cytokines ^26^. MNV-3 typically causes a sub-clinical persistent infection in wildtype (WT) C57BL/6 mice ^11^. Similarly, we did not observe any weight loss in miR-155 KO mice, indicating that there was no increase in disease severity (data not shown). Accordingly, MNV-3 was secreted at similar levels in the KO and WT mice throughout 21 days of a persistent infection ( Figure 3A). We harvested tissues at day 2 post infection to investigate if the lack of miR-155 affected dissemination of the virus, but we observed no difference in viral genome copies in the colon, caecum or ileum ( Figure 3B). Altogether this suggested that miR-155 does not play an essential role in controlling MNV replication in the acute or persistent phases of infection.

To determine whether miR-155 had any impact on the adaptive immune response to MNV-3, we compared development of the serum anti-MNV IgG response in infected miR-155 KO and WT mice. We found there was a lag in the production of serum IgG in the miR-155 KO mice, which had significantly lower levels compared to the WT mice at days 14, 21 and 28 hpi. Over this time course of the infection, the levels of serum IgG in the miR-155 KO mice did not reach those observed in WT mice.

## Discussion

In summary, we have identified a panel of miRNAs whose cell-type specific expression changes with MNV infection, indicating that MNV infection does not initiate a global shut-off in miRNA expression. miR-155 induction appears to be a marker of MNV infection in two permissive cell lines, as well as BMDMs and *in vivo* in infected tissues. However, the absence of miR-155 did not impact the course of infection or viral replication during persistent infection *in vivo*. This finding suggests that the immune defects associated with miR-155 deletion, such as lower serum IgG levels, are not important for control of persistent MNV infections.

miR-155 is one of the most highly studied miRNAs, upregulated in different cancers and with diverse cell-type specific roles reported ^27, 28^. In macrophage, miR-155 has been associated with the innate antiviral response, as it is induced as a result of signaling through the RIGI/JNK/NF-κB pathway ^24^. Upregulation of miR-155 promotes type I IFN signaling by silencing the negative regulator of the pathway, suppressor of cytokine signaling 1, (SOCS1) ^24^. Increased expression of miR-155 has also been shown to be accompanied by increased expression of pro-inflammatory mediators ^29^. As part of an inflammatory response the expression of miR-155 can also be increased indirectly through miR-342-5p ^29^, which was also induced in infected RAW264.7 cells and may have therefore contributed to the increase in miR-155 following MNV infection. As we observed no impact resulting from the loss of miR-155 on viral replication *in vivo,* in this study we did not further investigate which miR-155 targets are silenced in MNV-infected cells. A similar pro-inflammatory role has been demonstrated for miR146a in microglial cells ^30^, which we found was upregulated in BV-2 cells.

The defect we observed in the serum anti-MNV IgG response in miR-155 KO mice is consistent with their reported immune impairment, including defective TNFα production, reduced T-cell dependent antibody responses and a decrease in the proportion of INFγ-producing cells ^31^. Interestingly, this suggests that the serum IgG response and these other aspects of the immune response do not play a role in controlling persistent MNV infections *in vivo,* as despite the impaired responses, MNV replication was unaffected. This finding is in contrast to a previous report where antibody responses were proposed to contribute to clearance of acute infections, and the control of MNV replication in persistently infected mice, although this was performed using mice defective in B cells and RAG1 KO mice ^32^, suggesting this may be due to other defects in the antibody response than just serum IgG levels.

miR-687 was the most highly induced miRNA in both cell lines upon MNV infection, however very little is known about its function and expression profile. To date there has only been one study on the function of miR-687, which links it to regulation of cell cycle progression and apoptosis in kidney cells, through regulation of the phosphatase PTEN ^33^. PTEN has recently been reported to have a further independent function in regulating innate immunity, by controlling activation and nuclear import of the master transcription factor governing IFNβ production, IRF3 ^34^. Both apoptosis and IRF3 activation are thought to occur during MNV infection ^35, 36^, therefore whether miR-687 is involved in the regulation or crosstalk between these pathways will be an interesting avenue for future studies, which could reveal novel functions of miR-687 in the cellular response to viral stress.

In microglial BV-2 cells, downregulation of miR-17 was the greatest change associated with MNV infection. Further studies are required to validate this change in primary cells and infected tissues, but interestingly miR-17 has been shown to regulate autophagy through the suppression of Atg7 translation ^37^. Atg7 is known to be required for the INFγ antiviral response against MNV by promoting assembly of a complex of autophagy proteins, which in turn prevent formation of the MNV replication complex, although a direct mechanism linking INFγ and Atg7 has not been established ^38^. It is therefore interesting to speculate as to whether downregulation of miR-17 could provide this link, resulting in an increase in Atg7 translation, thereby contributing to the IFNγ antiviral response.

Finally, the recently developed organoid ^4^ and B cell culture systems ^5^ for HuNoV now provide the opportunity to compare the cell-type specific responses in miRNA expression, with the aim of identifying novel cellular proteins involved in HuNoV replication.

## Data availability

The raw data has been submitted to OSF http://doi.org/10.17605/OSF.IO/S85DY ^39^.

Data are available under the terms of the Creative Commons Zero "No rights reserved" data waiver (CC0 1.0 Public domain dedication).
