# Supplementary material for: miR-155 induction is a marker of murine norovirus infection but does not contribute to control of replication in vivo
Source: Wellcome Open Res. 2018 Apr 18;3:42. [Version 1] doi: 10.12688/wellcomeopenres.14188.1 (PMC5974592; doi:10.12688/wellcomeopenres.14188.1)
Supplement: Peer review history

# Peer review: Referee response for version 1

**DOI:** 10.21956/wellcomeopenres.15433.r33174

**Published:** 2018-05-29

**Authors:** Dirk Jochmans

**Affiliations:** 1 Laboratory of Virology and Chemotherapy, Department of Microbiology and Immunology, Rega Institute for Medical Research, KU Leuven, Leuven, Belgium

This manuscript by Thorne et al describes the first investigation on the role of miRNAs in norovirus infections. They discovered several miRNA’s of which the expression levels significantly change upon infection with MNV-1 in cell lines, which is a unique contribution to the field. They also show that a knockout of miR-155 does not influence the replication of MNV-3 in a mouse model. Both these results are sufficiently innovative to warrant the publication of this paper. In general the paper is well written and integrates very well the current knowledge on the topic.

 However there are two parts where we believe this paper could improve.

1. The cell line work has been done using MNV-1 while the animal work was performed with MNV-3. It is not impossible that the effect of norovirus on miR-155 is only apparent with MNV-1 and not with MNV-3. This would put the animal work in another perspective. It would therefore be very informative if the experiments described in fig 2.a, 2.b and 2.c are also performed with MNV-3. This may clearly demonstrate that also MNV-3 influences miR-155 expression *in vitro*. Now the only effect of MNV-3 on miR-155 expression is shown in fig 2.d but in our opinion, this is not sufficiently convincing to say that the effect is significant. In the first panel of fig 2.d the significance is indicated by “**” but it is not stipulated what the p value is here.
2. A second topic of discussion is the way the data is presented in figure 1. In our opinion, it is important to depict the fold change of expression level on a log scale. The expression levels are determined from Ct values that follow a normal distribution and for which a stdev can be calculated. If one log-transforms the Ct values to expression levels the normal distribution is lost and other statistics than stdev should be used. Therefore I would suggest that the authors calculate for each measurement the log(fold change over mock) and for these values calculate the average and stdev and show these on the y-axis of fig 1. This would also give a more clear presentation of the data around the 2-fold increase/decrease expression level. It would also benefit the reader significantly to plot the miR in a specific order and to put the expression of one miR in both cell lines next to each other. At this moment the reader cannot easily compare the expression levels of a particular miR between the cell lines.

In addition, we have some minor comments:

- In figure 1 the number of measurements are not indicated
- In the methods the manufacturer of the miRVana kit is not indicated
- In the first paragraph of the results section, the average values of the expression levels are indicated but not the statistics. An average of the log(fold change) and stdev could be indicated instead (see topic 2 above). The conclusion that miR-155 is upregulated in BV-2 cells is now difficult to conclude from fig 1.
- In the third paragraph of the result section the authors should refer to fig 2D
- In fig 3 LOD stands for limit of detection. Perhaps the authors mean limit of quantification?
- When referring to reference 32 in the conclusion the authors may want to comment that this was a paper studying MNV-1 and not MNV-3. It can be that IgG play a role in MNV-1 infections but not MNV-3 infections and vice versa for miR-155.

I have read this submission. I believe that I have an appropriate level of expertise to confirm that it is of an acceptable scientific standard.

# Peer review: Referee response for version 1

**DOI:** 10.21956/wellcomeopenres.15433.r33130

**Published:** 2018-05-21

**Authors:** Charlie C. Hsu

**Affiliations:** 1 Department of Comparative Medicine, University of Washington, Seattle, WA, USA

The manuscript “miR-155 induction is a marker of murine norovirus infection but does not contribute to control of replication *in vivo*” by Thorne et al. is a well written report describing the impact of MNV infection on microRNAs both *in vitro* and *in vivo*.  The reported findings are novel in that this is the first report evaluating the interaction of noroviruses and miRNAs.  The authors show that MNV indeed can modulate miRNA expression in cell lines, BMDM, and *in vivo*.  They report that miR-155, a microRNA involved with type I interferon signaling, is upregulated both *in vitro* and *in vivo*, but that miR-155 knockout mice did not show any differences in viral replication compared to infected wild-type mice despite having lower serum anti-MNV antibody levels.  These results suggest that miR-155 does not significantly contribute to control of MNV replication.  This manuscript is well organized and the authors do a nice job explaining their rationale for the experiments and they also thoughtfully discuss and interpret their results.

 This reviewer had only minor comments, suggestions, and thoughts:

- It is suggested to include the full nomenclature on the C57BL/6 wild-type mice, as well as the miR-155 knockout mice from Jackson Laboratories in the Methods to provide the readers information on which C57BL/6 substrain was used, and for accuracy of the miR-155 mouse strain.
- It would be interesting to see whether lack of miR-155 and the lower anti-MNV antibody levels in miR-155 knockout mice would impact viral levels and persistence in MNV-1, a typically non-persistent viral strain.  Although the authors provide an adequate explanation of why they used MNV-3, a persistent viral strain, for the *in vivo* study, rather than the MNV-1 that was used *in vitro*, this reviewer is curious if the lower antibody levels may perhaps prevent clearance of MNV-1 in these mice, or if virus persists longer than in WT mice but ultimately gets cleared.  These results may shed light on the importance (or lack of importance) of miR-155 associated immune defects on non-persistent viral strain.
- It is suggested to include information on the statistical tests used to evaluate differences if not already included in the manuscript.

I have read this submission. I believe that I have an appropriate level of expertise to confirm that it is of an acceptable scientific standard.
